# Field validation of secondary data sources: a novel measure of *representativity* applied to a Canadian food outlet database

**DOI:** 10.1186/1479-5868-10-77

**Published:** 2013-06-19

**Authors:** Christelle M Clary, Yan Kestens

**Affiliations:** 1 Social and Preventive Medicine Department, Université de Montréal, Montreal, Canada; 2 CRCHUM – Research Centre, Centre hospitalier de l’Université de Montréal, 3850 St-Urbain, Montreal, Quebec H2W 1T7, Canada

**Keywords:** Field validation, Food environment, Secondary database, Sensitivity, Positive predictive value, *Representativity*

## Abstract

**Background:**

Validation studies of secondary datasets used to characterize neighborhood food businesses generally evaluate how accurately the database represents the true situation on the ground. Depending on the research objectives, the characterization of the business environment may tolerate some inaccuracies (e.g. minor imprecisions in location or errors in business names). Furthermore, if the number of false negatives (FNs) and false positives (FPs) is balanced within a given area, one could argue that the database still provides a “fair” representation of existing resources in this area. Yet, *traditional* validation measures do not relax matching criteria, and treat FNs and FPs independently. Through the field validation of food businesses found in a Canadian database, this paper proposes alternative criteria for validity.

**Methods:**

Field validation of the 2010 Enhanced Points of Interest (EPOI) database (DMTI Spatial®) was performed in 2011 in 12 census tracts (CTs) in Montreal, Canada. Some 410 food outlets were extracted from the database and 484 were observed in the field. First, *traditional* measures of sensitivity and positive predictive value (PPV) accounting for every single mismatch between the field and the database were computed. Second, *relaxed* measures of sensitivity and PPV that tolerate mismatches in business names or slight imprecisions in location were assessed. A novel measure of *representativity* that further allows for compensation between FNs and FPs within the same business category and area was proposed. *Representativity* was computed at CT level as ((TPs +|FPs-FNs|)/(TPs+FNs)), with TPs meaning true positives, and |FPs-FNs| being the absolute value of the difference between the number of FNs and the number of FPs within each outlet category.

**Results:**

The EPOI database had a "moderate" capacity to detect an outlet present in the field (sensitivity: 54.5%) or to list only the outlets that actually existed in the field (PPV: 64.4%). *Relaxed* measures of sensitivity and PPV were respectively 65.5% and 77.3%. The *representativity* of the EPOI database was 77.7%.

**Conclusions:**

The novel measure of *representativity* might serve as an alternative to *traditional* validity measures, and could be more appropriate in certain situations, depending on the nature and scale of the research question.

## Background

Many studies have been performed to better understand the relationship between exposure to the foodscape – defined by Winson as “the multiplicity of sites where food is displayed for purchase and where it may also be consumed” [1] – and nutrition-related outcomes (e.g. obesity or dietary intakes) [2]. For pragmatic reasons, secondary data sources listing food outlets rather than field observations have been used to assess characteristics of the foodscape [3]. Uncertainty about the validity of such data sources raises the issue of potential and possibly systematic errors of measurement [4,5]. Recently, work has been conducted to validate commercial [6-9], Internet-derived [7,10] or government [8,10-12] databases, mainly in the US, the UK and Canada. Based on the match between database and field observation in “business name”, “category” or “location”, validity has traditionally been assessed using measures of sensitivity and positive predictive value (PPV), based on true positives (TPs), false positives (FPs) and false negatives (FNs). Variations in these metrics have been assessed over time [11], and in relation to neighborhood socioeconomic status [7,8,11,13,14], outlet type [6,13] or level of urbanization [8,13,14].

Criteria for validity are linked in some way to research objectives. For example, many studies have aimed to assess whether exposure - or access - to different types of food outlets influences nutrition-related outcomes [2]. Therefore, a database needs to provide a fair representation of the foodscape, i.e. an adequate evaluation of the number, type and localization of outlets. However, some slight differences between the database and reality may actually be very acceptable and have no impact on measures of foodscape exposure. For example, an error in the name of a business, when location and classification are correct, could be acceptable, since names of outlets are generally of secondary importance in studies that focus on foodscape influences. Similarly, if exposure is measured in terms of density (e.g. within a residential CT [15-18], home-centered buffer [19-21], or using kernel-density estimates [22]), a short distance between recorded and true locations should have little impact on the measure of exposure. This is particularly true if the “misplaced” outlet remains within the spatial unit in which density is computed. Furthermore, false positives (FPs) may be considered candidates for compensation for false negatives (FNs). For instance, if a database misses 10 outlets in a category in a given CT – 10 FNs - but at the same time lists 12 other outlets in the same category that are not present in the CT – 12 FPs - one can say that the database “overestimates” the number of outlets by only two. Yet, *traditional* measures of sensitivity and PPV apply distinctively to FPs and FNs and consider every single mismatch due to business name and location error. Such measures may underestimate the appropriateness of a database, for example when assessing foodscape exposure in terms of density within a specific area.

The present paper proposes a set of alternative validation measures for business listings while assessing the validity for research on the foodscape of the Enhanced Points of Interest (EPOI) file. EPOI is a Canadian database distributed by DMTI Spatial® (http://www.dmtispatial.com), containing over 1.6 million records of businesses. Validation was performed on food outlets listed in EPOI files within 12 CTs in Montreal. To our knowledge, no quantified validation study has been devoted to this sub-dataset, beyond minor reports of inconsistencies, missing data, or misclassifications [23].

In addition to *traditional* measures of sensitivity and PPV, we propose *relaxed* measures that tolerate mismatches in outlet names and within CT location errors. Furthermore, we introduce a novel measure of r*epresentativity* that allows for compensation between FPs and FNs within a given outlet category and CT. Variations in these measures are explored in relation to CT characteristics and outlet types.

## Methods

### Study area

Montreal (Island), Canada, is divided into 515 census tracts (CTs), each one covering an average surface of 0.9 km^2^ [min: 0.04 km^2^ – max 28.80 km^2^] and containing an average of 16.1 food outlets [min: 0 – max: 637]. Building on a previous validation project performed on a different database [7], the field validation occurred within 12 CTs in Montreal. Six CTs were predominantly French-speaking and six predominantly English-speaking. Within each language group, two CTs were sampled from each socioeconomic tertile (low, medium, high). Details about the CT sampling have been published elsewhere [7].

### Data sources

The list of food outlets was extracted from the EPOI dataset distributed by DMTI Spatial® and updated in 2010 (http://www.dmtispatial.com). For each listed outlet, the database provides a name, a postal address, a geographic coordinate, and between one and six Standard Industrial Classification (SIC) codes (four characters long), assigned to a business based on the economic activities it declares [24]. SIC codes are increasingly being replaced by the North American Industry Classification System (NAICS), which provides more specific codes (http://www.census.gov/eos/www/naics/), but is not available in this database.

### Classification of food outlets

We defined 11 categories of food outlets, eight of which were food stores - mega-markets, chain supermarkets, grocery stores, convenience stores, bakery shops, fruit and vegetable stores, specialty markets (e.g. butcher or cheese shops), natural food stores - and three food services - fast-food restaurants, full-service restaurants and cafés. Establishments that were primarily bars, liquor stores or caterers were not retained. SIC codes offer a rough classification, some codes encompassing quite different types of outlets (e.g. SIC code “5411” includes mega-markets, chain supermarkets, and grocery stores as well as convenience stores). To assign food outlets to a given category, categorization was based on a SIC code- and name-based assignment method, relying upon the researcher’s knowledge of the local food environment. Details on this method are shown in Additional file [see Additional file 1]. In short, we first extracted all outlets engaged in the retail of foods (SIC codes “5411 – grocery stores”; “5421 – meat and fish markets”; “5431 – fruit and vegetable markets”; “5441 – candy, nuts and confectionary stores”; “5451 – dairy product stores”; “5461 – retail bakeries”; and “5499 – miscellaneous food stores”), as well as eating places (SIC code “5812”) and drinking places (SIC code “5813”). Second, outlet categories were identified using both requests on SIC code and keyword requests on business name. For instance, convenience stores were outlets with a SIC code starting with “54” and a business name having at least one keyword alluding to this outlet category (e.g. “convenience”, “convenient”, “gas station”, etc.), including brand name (e.g. “Bonisoir”, “Couche-Tard”, etc.). Similarly, chain supermarkets were identified as outlets with a SIC code starting with “54” and having a supermarket brand business name (e.g. “Provigo”, “Metro”, “IGA”, etc.). Because an outlet can declare up to six SIC codes to portray its overall activities, it could potentially have been included in more than one category. To avoid such duplicate affiliations, outlets were not made available to another category once extracted. For example, an outlet called “Provigo” declaring SIC codes “5411” (grocery), “5461” (dairy products), “5431” (fruit and vegetable) and “5421” (fish and meat) was assigned to the “chain supermarkets” category and not made available to any other categories such as “fruit and vegetable stores” or “specialty market”. After identifying all outlets that came under one of the 11 defined categories, those located within the 12 targeted CTs were retained for field validation. The resulting list of outlets was reviewed, and duplicate entries based on both names and street addresses were removed. Records displaying strictly identical street addresses but names that differed only due to an additional reference to an administrative function (e.g. “office”, “fax”) were considered duplicates.

### Field validation

One observer undertook field validation on foot in the daytime over a two-week period in October 2011, following a one-day training period during which the observer’s recordings were verified using a testing CT. Supplied with EPOI lists for the 12 CTs, this person identified unlisted businesses found in the field and listed food stores trading under a different name, at a different address, or falling into a different category. An additional file shows the classification rules used to categorize observed food outlets [see Additional file 2]. Outlets that appeared to be closed permanently were not considered to be present in the field. Outlets found in the field but not listed in the database were manually searched in the whole EPOI database, using the business name and street address. This allowed further identification of FNs that would be listed in the EPOI but incorrectly geocoded outside of the selected CT. Such observations were classified as "ill-extracted", i.e. present in the whole database under the right name and street address, but wrongly geocoded. Inversely, the address and geographic coordinates of FPs were checked to ensure that the outlet had not been "inappropriately included" due to geocoding errors.

### Data analysis

Firstly, the overall validity of the EPOI database was quantified through *traditional* measures of sensitivity and PPV, while considering errors in “name”, “location” or “categorization” as mismatches (cf. Table 1). Second, *relaxed* measures of sensitivity and PPV were computed. These ignored mismatches due to a difference in outlet name or to a within-CT inaccuracy in location (e.g. listed outlet with wrong address but correctly in the CT). Third, a novel measure of *representativity* was proposed as follows:

$$\mathrm{Representativity}=\left({}^{\left(\mathrm{TPs}+|\mathrm{FPs}-\mathrm{FNs}|\right)}/{}_{\left(\mathrm{TPs}+\mathrm{FNs}\right)}\right)$$

with TPs meaning true positives, and |FPs-FNs| being the absolute value of the difference between the number of FNs and the number of FPs within each outlet category.

**Table 1:** **Calculation of *traditional *and *relaxed *sensitivity and positive predictive value (PPV), and *representativity***

	**Outlet present (field)**		**Outlet absent (field)**	
Outlet present (database)	True positive (TP)		False positive (FP)	
	*For traditional measures*	*For relaxed measures/ Representativity*	*For traditional measures*	*For relaxed measures/ Representativity*
	Match with respect to any name, location and category error	Match disregarding errors in name and imprecisions in location	Includes outlets mismatched due to name errors and imprecisions in location	Excluding outlets mismatched due to name errors and imprecisions in location
Outlet absent (database)	False negative (FN)		True negative (TN)	
	*For traditional measures*	*For relaxed measures/ Representativity*		
	Including outlets mismatched due to name errors and imprecisions in location	Excluding outlets mismatched due to name errors and imprecisions in location		

Measures of sensitivity, PPV and *representativity* were computed for each of the 12 CTs. Overall values for these metrics were computed as the average of all CT-level measures, weighted by the number of outlets per CT. Measures below 0.30 were considered as "poor", from 0.31–0.50 as "fair", from 0.51–0.70 as "moderate", from 0.71–0.90 as "good", and over 0.90 as "excellent". Such a scale is only provided for indicative purposes, as terminology can be debatable (e.g. "good", with a value of 0.71, fails to identify an existing outlet or identifies a non-existent one about one-third of the time). These descriptors were adopted, however, for the purpose of more easily comparing results with the existing literature [7,10]. Pearson’s chi-square tests of independence performed with SPSS were used to assess variations in sensitivity and PPV in relation to CT socioeconomic status (“low”, “medium”, “high”), CT language (“French”, “English”) and outlet category. Sensitivity and PPV were displayed in contingency tables as binary variables. Sensitivity was said to be "not encountered" when an outlet was present in the field but not on the list (false negative), and "encountered" when an outlet was both present in the field and listed (true positive). Similarly, PPV was said to be "not encountered" when an outlet was present on the list but not in the field (false positive), and "encountered" when an outlet was both present in the field and listed (true positive). In order to reach a critical size per cell, mega-markets and chain supermarkets were combined. Although the conditional Fisher’s exact test (two-sided p) has been widely used to assess such variations [7,8,11,13,14], we do not recommend it. Primarily, the expected "beforehand fixed margins" condition (i.e. the row sums and the column sums are fixed prior to the study) is not encountered in observational studies [25]. In fact, the number of outlets – whether correctly listed (TPs), listed but not found in the field (FPs), or not listed but found in the field (FNs) – can only be deduced from field observations. Some have therefore suggested that this test "should practically never be used" [26].

## Results

After removing 22 duplicate entries, the EPOI database provided a list of 410 outlets, of which 50.0% were full-service restaurants, 12.4% convenience stores, 9.0% cafés, 7.8% fast-food restaurants, 7.1% grocery stores, 5.1% bakeries, 3.2% specialty markets, 2.2% fruit and vegetable stores, 2.2% natural food stores, 0.7% chain supermarkets, and 0.2% mega-markets (Table 2).

**Table 2:** Records in EPOI database against field observations

		**No. of outlets listed**	**Disposition**						**No. of outlets found but not listed**	
			**Matching**	**Not matching**				**Not found**		
				**Error in name**	**Error in location**	**Error in category**	**Error in both name and category**		**Not present in EPOI database**	**Ill-extracted from EPOI database**
	**Total**	**410**	**264**	**50**	**3**	**16**	**12**	**65**	**105**	**34**
Food stores	Convenience stores	51	37	9	1	1	1	2	13	1
	Chain supermarkets	3	3	0	0	0	0	0	1	0
	Grocery stores	29	16	0	0	6	2	5	5	4
	Bakeries	21	13	2	0	3	0	3	6	3
	Specialty markets	13	9	0	0	0	0	4	4	1
	Fruit and vegetable stores	9	5	2	0	0	0	2	1	3
	Natural food stores	9	7	0	0	1	0	1	6	2
	Megamarkets	1	1	0	0	0	0	0	0	0
Food services	Fast-food restaurants	32	25	0	1	1	2	3	11	2
	Cafés	37	18	1	1	2	3	12	19	6
	Full-service restaurants	205	130	36	0	2	4	33	39	12
Census tract predominant LANGUAGE	English	295	186	41	3	13	9	43	75	22
	French	115	78	9	0	3	3	22	30	12
Census tract SES	Low	73	51	5	2	1	0	14	22	9
	Medium	164	102	29	1	7	6	19	41	6
	HIGH	173	111	16	0	8	6	32	42	19

The fieldwork recorded a total of 484 outlets. Of the 410 listed outlets, 264 matched perfectly with the outlets observed in the field, while 81 were mismatched, including 50 mismatched in “name”, 3 in “location”, 16 in “category” and 12 in both “name” and “category”. Some 139 outlets found in the field were not listed in the extracted list. Of these, 34 were actually present in the remaining records of the complete EPOI database. While their names, categories and street addresses were correctly documented, a geocoding error, probably associated with an error in the 6-digit postal code, had prevented their correct spatial location and corresponding extraction. Some 65 listed outlets were not found in the field. None owed their erroneous presence to geocoding errors, as their street addresses were located in the appropriate CT. However, the EPOI database shows a significant number of geocoding inaccuracies. For the entire set of Montreal food outlets (n=8300), 6.9% of outlets (n=570) had a poor geocoding precision code (i.e. geocoded at municipal centroid).

### *Traditional* and *relaxed* measures of sensitivity and PPV

*Traditional* sensitivity was 54.5% (CI [48.7% - 60.3%]), and PPV 64.4% (CI [59.2% - 69.6%]), or “moderate” (Table 3). When relaxing matching criteria on “name” or “location”, sensitivity increased to 65.5% (CI [59.2% - 71.8%]) and PPV to 77.3% (CI [73.6% - 81.0%]).

**Table 3:** Validation statistics for the EPOI database

				***Traditional *measures**				***Relaxed *measures**					
	**N Total**	**N List**	**N Field**	**Sensitivity**		**Positive predictive value**		***Relaxed *sensitivity**		***Relaxed *positive predictive value**		***Representativity***	
				**Est.**		**Est.**		**Est.**		**Est.**		**Est.**	
Overall	549	410	484	0.545		0.644		0.655		0.773		0.777	
				0.487	0.603	0.592	0.696	0.592	0.718	0.736	0.810	0.713	0.840
Census tract characteristics													
Low SES	104	73	90	0.567		0.699		0.700		0.732		0.756	
				0.508	0.625	0.590	0.808	0.478	0.923	0.581	0.882	0.723	0.788
Medium SES	211	164	192	0.531		0.622		0.748		0.830		0.797	
				0.454	0.608	0.515	0.729	0.578	0.917	0.788	0.871	0.753	0.841
High SES	234	173	202	0.550		0.642		0.578		0.709		0.762	
				0.422	0.677	0.605	0.678	0.394	0.761	0.660	0.758	0.628	0.897
English	392	295	349	0.533		0.631		0.690		0.755		0.791	
				0.472	0.594	0.567	0.694	0.544	0.837	0.713	0.798	0.740	0.842
French	157	115	135	0.578		0.678		0.660		0.758		0.733	
				0.462	0.693	0.592	0.764	0.485	0.835	0.644	0.873	0.633	0.833
Categories of outlet													
Convenience store	65	51	63	0.587		0.725		0.746		0.922		0.762	
				0.489	0.686	0.604	0.847	0.636	0.856	0.856	0.987	0.644	0.880
Chain supermarkets	4	3	4	0.750		1.000		0.750		1.000		0.750	
				0.325	1.175	1.000	1.000	0.325	1.175	1.000	1.000	0.325	1.175
Grocery stores	38	29	33	0.485		0.552		0.485		0.552		0.545	
				0.278	0.692	0.371	0.732	0.278	0.692	0.371	0.732	0.361	0.730
Bakeries	30	21	27	0.481		0.619		0.556		0.714		0.593	
				0.289	0.674	0.428	0.810	0.320	0.791	0.512	0.916	0.338	0.847
Specialty markets	18	13	14	0.643		0.692		0.643		0.692		0.786	
				0.423	0.863	0.563	0.822	0.423	0.863	0.563	0.822	0.539	1.032
Fruit and vegetable stores	13	9	11	0.455		0.556		0.636		0.778		0.727	
				0.170	0.740	0.304	0.807	0.353	0.920	0.502	1.053	0.414	1.040
Natural food stores	17	9	16	0.438		0.778		0.438		0.778		0.500	
				0.141	0.734	0.413	1.142	0.141	0.734	0.413	1.142	0.200	0.800
Megamarkets	1	1	1	1.000		1.000		1.000		1.000		1.000	
Fast-food restaurants	45	32	42	0.595		0.781		0.619		0.813		0.690	
				0.459	0.731	0.707	0.856	0.486	0.752	0.747	0.878	0.533	0.848
Cafés	62	37	50	0.360		0.486		0.400		0.541		0.600	
				0.249	0.471	0.339	0.634	0.297	0.503	0.421	0.660	0.453	0.747
Full-service restaurants	256	205	223	0.583		0.634		0.744		0.810		0.861	
				0.522	0.644	0.581	0.687	0.690	0.799	0.770	0.850	0.809	0.913

### Novel measure of *representativity*

Further accounting for the compensation between FNs and FPs provided a “good” *representativity* measure of 77.7%; (CI [71.3% - 84.0%]).

### Variations

No significant difference was observed by CT characteristic (SES and language) for both *traditional* and *relaxed* measures. Chi-square analyses indicated no between-category differences in *traditional* sensitivity (Pearson Chi Square’s *p = 0.413*) or PPV (*p* = 0.058). Significant differences were, however, observed for *relaxed* sensitivity (*p = 0.001*) and PPV (*p = 0.000*), with higher values obtained for convenience stores, full-service restaurants, and fruit and vegetable stores compared to other outlets.

## Discussion

Secondary data sources offer various options for describing foodscapes. Yet the validity of such commercial, government and Internet-derived database needs to be evaluated. This paper assessed the validity of the EPOI database in 12 CTs in Montreal, Canada. *Relaxed* measures of sensitivity and PPV were compared to *traditional* measures, and a novel measure of *representativity* was proposed. *Traditional* validity measures indicated a "moderate" capacity of the database to detect the presence of outlets in the field (sensitivity of 54.5%; CI [48.7% - 60.3%]) or list the sole outlets actually existing in the field (PPV of 64.4%; CI [59.2% - 69.6%]). No evidence of systematic differences related to CT characteristics or outlet category was observed. These findings are similar to others previously reported in the literature, in the "fair" to "moderate" range [8,13], although some studies have reported sensitivity and PPV in the "good" to "excellent" range [7,11,14]. How do these results help us to reach conclusions about the appropriateness of such a database for evaluating foodscapes, however?

The question of what criteria should prevail in order to consider a database "valid" for foodscape characterization has not been much debated. Whereas in some studies any difference in “name”, “location” or “category” is considered a mismatch [8,13], others relax on certain criteria – including name, address, category, or subcategory [7,11,13]. Our findings showed that measures of sensitivity and PPV do differ quite substantially on whether or not they ignore name errors or inaccuracies in location. Estimates of sensitivity and PPV respectively increased from 54.5% and 64.4% to 65.5% and 77.3% after relaxing on those aspects. Differences in the choice of matching criteria may partly explain why some studies have concluded that secondary data sources provide a valid alternative to fieldwork [7,9,14], while others have expressed the need for caution [8,10,13].

Such discrepancies raise the issue of which criteria should be considered to assess the validity of databases for use in characterizing foodscapes. Because exposure is often measured in terms of density for a given outlet type at a given location (e.g. within the residential CT, within a home- or school-centered buffer, or using kernel estimates), discrepancies in business names or small location errors (e.g. records staying in the same spatial unit) have no impact on exposure estimates. Whether the database is an exact copy of the field may not be relevant. *Traditional* measures of validity that account for every single mismatch in business name or exact location may be too conservative and lead to misguided recommendations for use. Along these lines, FPs and FNs should not always be considered independently, but rather seen as a whole. Some have advised combining multiple data sets to reduce FNs and increase PPV [10]. Such a strategy may, however, inappropriately increase the number of FPs and decrease sensitivity [8]. The criteria that should prevail to determine whether or not an observed difference between the database and the field is acceptable should vary according to the research objectives. The proposed measure of *representativity* allows for compensation between FPs and FNs, while errors in business names or minor location inaccuracies can be tolerated. When the dataset is used to assess densities of outlets, *representativity* offers a good complement to *traditional* validity measures. Yet, when relaxing on location and offsetting FNs with FPs, a “spatial tolerance threshold” must be set. This threshold can be of the form “must stay within a same spatial unit” or “must stay within a given distance”. Consequently, *relaxed* measures of validity – allowing spatial imprecision – and *representativity* – allowing compensation between FPs and FNs – are dependent on these spatial criteria. Smaller tolerance thresholds – say, location errors of less than 100 meters, or compensation between FPs and FNs only allowed within a short distance from each other or within the same small spatial unit – are less permissive. Measures of *representativity* should therefore always be provided along with a spatial tolerance criterion. If an exact representation of the field is needed, *relaxed* or *representativity* measures are not useful. This may be the case when databases are used to obtain exact measures of proximity (e.g. [27-29]). Therefore, we do not recommend systematic reliance on *representativity*. We believe it is an interesting metric to document how close a database is able to “represent” a true measure of exposure. The relevance and appropriateness of this *representativity* does, however, depend on the research objectives and methods used to assess exposures.

With a *representativity* of 77.7% (CI [71.3% - 84.0%]), the EPOI database represents 77.7% of the CT foodscape, which can be considered good but not excellent. Correcting the 34 geocoding errors raises *representativity* to 80.5% (CI [74.2% - 86.7%], which shows how deleterious geocoding inaccuracies can be [30], but they can also be identified and sometimes corrected. Specifically, one needs to scan such a database to assess unique coordinate frequencies and detect possible artificial clusters due to geocoding approximations. In Montreal, a large number of outlets coded at the city level would fall within a single CT, for which the density estimate is consequently extremely inaccurate. Refining geocoding constitutes an interesting avenue for improving the quality of the EPOI database.

### Limitations

Firstly, because validation measures were limited to 12 CTs (i.e. 2.3% of all CTs in Montreal), cautious interpretation is required. The small size of our sample may have resulted in unstable estimates of error. Overall patterns were consistent along Montreal’s urban socioeconomic and language composition, suggesting that our estimates are reasonably reliable and valid. Yet, those variations were tested based on only two CTs in each SES and language category. Therefore, the design of our study may have lacked the power to detect them. Further validation in different cities and using wider data samples would be useful to allow for generalizability. Particular caution should be expressed regarding rural areas, as our sample did not cover that type of territory.

Second, because the field validation occurred one year after the EPOI dataset was released, actual changes in the foodscape [31-33] may have affected validity measures. Outlets closing, opening, rebranding, and changes of ownership over this one-year time-lapse have presumably increased the number of FPs, FNs and mismatches in business name. This impact potentially contributed to underestimating both *traditional* and *relaxed* measures of validity. Since *relaxed* measures overlook mismatches in outlet name, the one-year time-lapse should, however, have a lower impact on *relaxed* than on *traditional* measures. Additionally to this quantitative aspect, the way the foodscape renewed itself over this period of time may also have qualitative implications. For instance, areas with major arrivals of new migrants may experience modifications in the nature of the food offer (e.g. closing of six convenience stores and opening of nine specialty outlets or ethnic stores over the same time period). Inversely, in areas with a stable socio-demographic structure, the food offer may stay roughly the same (e.g. closing of six convenience stores followed by the opening of five new convenience stores). In the former case, the possibility of compensation is null, while in the latter, five compensations for FPs by FNs are made possible. However, since we know little about foodscape dynamics over the one-year time-lapse that separates the EPOI database release from field validation, we cannot say how much this period of time has specifically affected *representativity*.

Third, since some head offices operate under a different name than the attached retail outlet, some of the duplicate entries we aimed at removing may have been overlooked. If such head offices had been purged back, the performance of the EPOI database could have been improved.

Finally, the method we chose to categorize outlets may have led to some misclassifications. The name-based assignment method used to compensate for low-specific SIC codes may have failed to assign some outlets to the correct category, or the observer may have assigned the wrong category to a given outlet observed in the field. Despite some attempts [10,34], no precise criteria were agreed upon for rigorously and systematically assigning an outlet to a given category. We proposed a name-based assignment method to refine the EPOI database categorization, and the observer was equipped with a classification tool that helped categorize outlets based on the type, nutritional quality and specificity of their offerings, as well as the size of the premises [see Additional File 2]. However, the wide-ranging activity of some outlets (e.g. restaurants that also offer take-out food, or supermarkets that have counters with food for on-site consumption), may have made exclusive classification difficult. Along these lines, as most outlets sell both healthy and unhealthy options, though in different proportions, the right assignment is made difficult (e.g. distinction between convenience and grocery stores).

Because the observer was not blinded to the EPOI list during field validation, he may have been tempted to adopt the EPOI list’s categorization when the assignment of an outlet to a single category based on field observation was difficult. Therefore, the number of category errors identified (n=16 out of 410 outlets listed) might have been underestimated, and validity measures overestimated. Further research should provide better guidelines for classifying food outlets. Such criteria should guide correspondence between commercial classifications of outlets as they appear in secondary data sources, and classifications according to the nutritional behavior they promote. The multiple nature of some outlets (e.g. with both food for on-site consumption and take-out food, or with both healthy and unhealthy offerings) remains a challenge for assessing exposure.

## Conclusions

It is important to assess the validity of secondary databases used to characterize foodscapes in order to obtain valid estimates of exposure and reduce bias. The proposed measures of *relaxed* sensitivity and PPV, and particularly the novel measure of *representativity,* offer interesting alternatives to *traditional* measures of validity. The EPOI database had a poor capacity to detect the exact outlets in the field. However, relaxing on outlet names and allowing small location imprecisions improves its performance. Furthermore, when compensation between FPs and FNs was allowed within CTs, the EPOI database offered good *representativity* of the CT foodscape. The EPOI database can consequently be considered as inadequate for measuring exact distance to specific outlets, but it is a valuable resource for assessing local densities. Therefore, it is not so much which of *traditional* or *relaxed* measures are “superior”, as under what circumstances the use of *relaxed* and *representativity* measures may be more appropriate.

## Abbreviations

CT: Census tract; EPOI: Enhanced Points of Interest; FNs: False negatives; FPs: False positives; NAICS: North American Industry Classification System; PPV: Positive predictive value; SES: Socioeconomic status; SIC: Standard Industrial Classification; TNs: True negatives; TPs: True positives.

## Competing interests

Christelle M Clary has no financial disclosure. Yan Kestens has no financial disclosure.

## Authors’ contributions

CC carried out the field validation study, organized the one-day training period for the field observer, performed the statistical analyses and drafted the manuscript. YK participated in the design of the study, contributed to the statistical analyses and revised the manuscript. Both authors approved the final manuscript.

## Supplementary Material

Additional file 1 SIC codes- and name-based assignment method used to categorize food outlets. Click here for file

Additional file 2 Classification tool aiming at facilitating categorization of food outlets found on-site. Click here for file
