# Supplementary material for: Field validation of secondary data sources: a novel measure of representativity applied to a Canadian food outlet database
Source: Int J Behav Nutr Phys Act. 2013 Jun 19;10:77. doi: 10.1186/1479-5868-10-77 (PMC3710283; doi:10.1186/1479-5868-10-77)
Supplement: Additional file 1 — SIC codes- and name-based assignment method used to categorize food outlets. [file 1479-5868-10-77-S1.doc]

Additional file 1: SIC codes- and name-based assignment method used to categorize food outlets.

| **Outlet category** | **Definition** | **Assignment method** | **Dataset used for extraction** | |  |
| --- | --- | --- | --- | --- | --- |
| All food outlets together | Business selling food as their primary activity or as a secondary activity | **Request on SIC codes**  Outlets with at least one of the following SIC codes:  - “5411” - Grocery stores defined as stores engaged in the retail sale of all sorts of canned and dry goods, fruits and vegetables, and fresh and prepared meals  - “5421” - Meat and fish markets defined as establishments engaged in the retail sale of fresh, frozen or cured meats, fish, shellfish, and other seafood,  - “5431” - Fruit and vegetable markets defined as establishments engaged in the retail sale of fresh fruits and vegetables  - “5441” - Candy, nut and confectionery stores defined as establishments engaged in the retail sale of candy, nuts, popcorn and other confections,  - “5451” - Dairy product stores defined as establishments engaged in the retail sale of packaged dairy products  - “5461” - Retail bakeries defined as establishments engaged in the retail sale of bakery products  - “5499” - Miscellaneous food stores defined as establishments engaged in the retail sale of specialized foods  - “5812” - Eating places defined as establishments engaged in the retail sale of prepared food and drinks  - “5813” - Drinking places defined as establishments engaged in the retail sale of alcoholic drinks | Original EPOI dataset | |  |
| Convenience stores | Local food stores selling a limited range of fresh and healthy products like fruits and vegetables | **Request on both SIC codes and business name**  Outlets with a SIC code starting with “54” AND a business name with at least one keyword alluding to this outlet category, including brand name (“convenience”, “convenient”, “petrol station”, “Bonisoir”, “Couche tard”, etc.). | Dataset 0, i.e. initial dataset encompassing all food outlets | |  |
| Chain supermarkets | Large retail food store chains selling a wide range of fresh and healthy products like fruits and vegetables | **Request on both SIC codes and business name**  Outlets having a SIC code starting with “54” AND a supermarket brand business name (“Provigo”, “Metro”, “IGA”, etc.) | Dataset 1, i.e. dataset 0 without convenience stores | |  |
| Mega-markets | Large retail food store chains offering a wide range of fresh and healthy products like fruit and vegetable, and non-food products | **Request on both SIC codes and business name**  Outlets having a SIC code starting with “54” AND a mega-market brand business name (“Loblaw”, “Maxi”, “Super C”, etc.) | Dataset 2, i.e. dataset 1 without chain supermarkets | |  |
| Fruit and vegetable stores | Food stores specialized in the retail of fruits and vegetables | **Request on both SIC codes and business name**  Outlets with a SIC code “5431”, AND a business name NOT with keywords alluding to other outlets’ categories (“grocery”, “bread”, “fish”, “meat”, “cheese”, etc.) | Dataset 3, i.e. dataset 2 without mega-markets | |  |
| Bakeries | Food stores specialized in the retail of bread and other baked products (includes bagel shop) | **Request on both SIC codes and business name**  Outlets with a SIC code “5461”, AND a business name NOT with keywords alluding to other outlets’ categories (“grocery”, “fish”, “meat”, “cheese”, “cafe”, “muffin”, “doughnut”, etc.) | Dataset 4, i.e. dataset 3 without fruit and vegetable stores | |  |
| Specialty stores | Food stores specialized in the retail of meat, cheese, or fish and other seafood | **Request on both SIC codes and business name**  Outlets with a SIC code “5421”, AND a business name NOT with keywords alluding to other outlets’ categories (“grocery”, “cafes”, etc.).  OR  Outlets with a SIC code starting with “54” AND a business name with at least one keyword alluding to this outlet category (“fish”, “meat”, “cheese”, “butcher”, etc.) | Dataset 5, i.e. dataset 4 without bakeries | |  |
| Natural food stores | Food stores specialized in the retail of natural food, organic food, or food supplements | **Request on both SIC codes and business name**  Outlets with a SIC code “5499” AND a business name with at least one keyword of a list alluding to this outlet category (“nature”, “health”, “organic”, “life”, “herb”, “garden”, etc.) | Dataset 6, i.e. dataset 5 without specialty stores | |  |
| Grocery stores | Local food stores selling a wide range of fresh and healthy products (e.g. fruit and vegetable), and/or ethnic foods | **Request on both SIC codes and business name**  Outlets with a SIC code “5411” AND a business name NOT with keywords alluding to other outlets’ categories (“caterer”, “restaurant”, “cafe”, etc.)  OR  Outlets with a SIC code “5421” or “5431” or “5451” or “5461” or “5499”, AND a business name with at least one keyword alluding to this outlet category (“grocery”, “grocer”, “market”, “shop”, etc.) | Dataset 7, i.e. dataset 6 without natural food stores | |  |
| Fast-food restaurants | Chain or independent outlets selling ready-to-eat food - mainly high energy density foods - with a counter service | **Request on business name**  Outlets with a business name with at least one keyword alluding to this outlet category, including brand name (“burger”, “fried”, “2 for 1”, “hot dog”, “A&W”, “McDonald”, “KFC-PKF”, etc.) | Dataset 8, i.e. dataset 7 without grocery stores | |  |
| Cafes | Chain or independent outlets specialized in snack foods to have in or to take away (includes juice bars, ice-cream shops and cake/pastry shops like “MMMuffins”) | **Request on both SIC codes and business name**  Outlets with a SIC code “5812” or “5813” or “5461” or “5499”, AND a business name with at least one keyword alluding to this outlet category, including brand name (“café”, “Starbuck”, “Tim Horton”, “Van Houtte”, “MMMuffins”, “doughnut”), AND not with keywords referring to other outlets’ categories like “caterer” and “restaurant” | Dataset 9, i.e. dataset 8 without fast-food restaurants | |  |
| Full-service restaurants | Chain or independent outlets offering a wide range of ready-to-eat food with table service. | **Request on both SIC codes and business name**  Outlets with a SIC code “5812”, AND a business name NOT with any keywords alluding to other outlets’ categories (“caterer”, “sugar house”, etc.)  OR  Outlets with a SIC code “5813”, AND a business name with at least one keyword alluding to this outlet category (e.g. “restaurant”, “bistro”, “brasserie”, etc.) | Dataset 10, i.e. dataset 9 without cafes | |  |
| Others  (Liquor stores, bar, pub, caterer, etc.) |  | Residual dataset was checked and requests were refined until only bars, liquor shops, caterer, and other out-of-scope outlets left as residuals. | | Residual dataset, i.e. dataset 10 without restaurants | |
