# Supplementary material for: Field validation of secondary data sources: a novel measure of representativity applied to a Canadian food outlet database
Source: Int J Behav Nutr Phys Act. 2013 Jun 19;10:77. doi: 10.1186/1479-5868-10-77 (PMC3710283; doi:10.1186/1479-5868-10-77)

Additional file 2: Classification tool aiming at facilitating categorization of food outlets found on-site

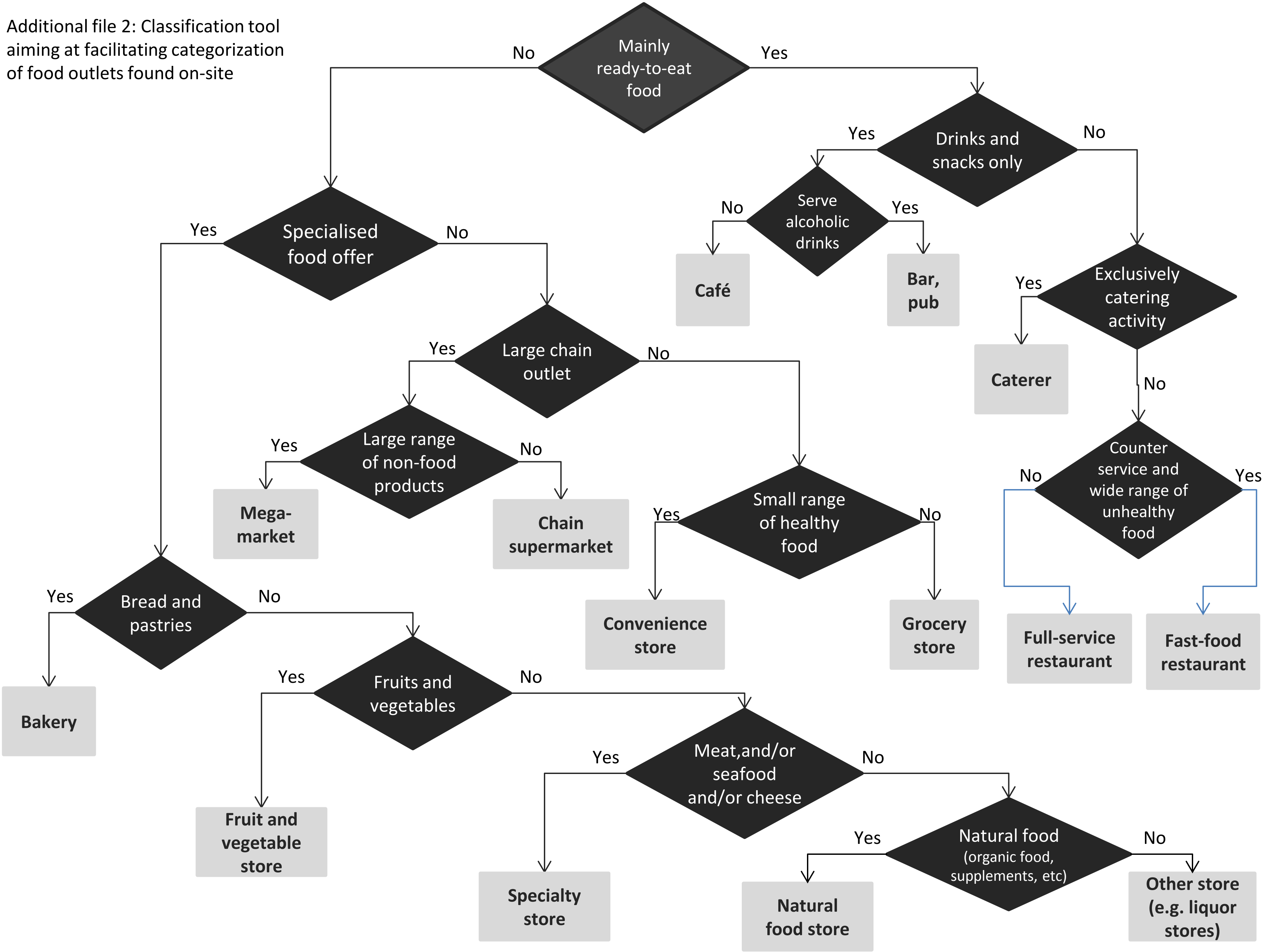

Supplement: Additional file 2 — Classification tool aiming at facilitating categorization of food outlets found on-site. [file 1479-5868-10-77-S2.pdf]
